# What are we teaching UK medical students about ageing and geriatric medicine? Results of the third British Geriatrics Society national curriculum and teaching survey

**DOI:** 10.1093/ageing/afaf216

**Published:** 2025-08-11

**Authors:** Alice Watson, Grace Margaret Evelyn Pearson, Rebecca Winter, Adrian Blundell, Tahir Masud, Adam Lee Gordon, Emily Jane Henderson

**Affiliations:** Bristol Medical School, University of Bristol, Bristol, UK; Royal Devon University Healthcare NHS Foundation Trust, Exeter, UK; Bristol Medical School, University of Bristol, Bristol, UK; Older Persons Unit, Royal United Hospitals Bath NHS Foundation Trust, Bath, UK; Medical Education, Brighton and Sussex Medical School, Brighton, UK; Healthcare of the Older Person, Nottingham University Hospitals NHS Trust, Nottingham, UK; Faculty of Medicine and Health Sciences, University of Nottingham, Nottingham, UK; Faculty of Medicine and Health Sciences, University of Nottingham, Nottingham, UK; Healthcare for Older People, Nottingham University Hospitals NHS Trust, Nottingham, UK; Wolfson Institute of Population Health, Queen Mary University of London, London, UK; Academic Centre for Healthy Ageing, Barts Health NHS Trust, London, UK; Bristol Medical School, University of Bristol, Bristol, UK; Older Persons Unit, Royal United Hospitals Bath NHS Foundation Trust, Bath, UK

**Keywords:** undergraduate education, medical education, ageing education, teaching survey, curriculum survey, older people

## Abstract

**Introduction:**

The ageing population means all newly qualified doctors will need knowledge, skills and attitudes to care for older people with complex health conditions. An essential component of preparing the medical workforce to care for older people is including teaching on ageing and geriatric medicine in undergraduate medical curricula. We present results of the British Geriatrics Society national curriculum survey 2021–22, highlighting progress in undergraduate teaching in geriatric medicine.

**Methods:**

All 35 UK General Medical Council–registered medical schools at the time of data collection were invited to participate in an online survey on the content, methodology, timing and duration of teaching in ageing and geriatric medicine.

**Results:**

Thirty out of the thirty-five (83%) UK medical schools responded, all of which provided some teaching in geriatric medicine. Most teaching occurred in the fourth year of study (*n* = 21). The majority (*n* = 15) reported a discrete module for geriatric medicine lasting 4–8 weeks, an increase on previous surveys. Three schools reported geriatric medicine exposure lasting >12 weeks, of which two were integrated clerkships. We observed a reduction in interprofessional education but identified several examples combining simulated teaching with other healthcare professions (*n* = 7). Every school (*n* = 30) incorporated small-group or case-based learning.

**Conclusion:**

There is increased exposure to geriatric medicine compared to previous surveys in 2008 and 2013. Generally, programmes reported a move towards clinically framed small-group and case-based learning, employing a wider variety of assessment methods than previously. We also identified key gaps in teaching provision for targeted improvement and ongoing research.

## Key Points

- Our national survey of undergraduate teaching in ageing and geriatrics has identified improvements in coverage and duration.
- There has been a shift towards small-group and case-based learning, and a wider variety of assessment methods are in use.
- Some topics are still poorly covered and there was a decrease in interprofessional learning, which are key areas for future focus.
- Exemplar teaching practices included interprofessional simulation and involvement of community-dwelling older people.
- Together with the British Geriatrics Society recommended undergraduate curriculum, our results highlight models for best practice in ageing education.

## Introduction

The World Health Organisation (WHO) predicts that, in 2030, 1 in 6 people will be over the age of 60 and that by 2050 the number of people aged 80 and older will have tripled [1]. Ageing carries with it complexities in the health and care of older people [2]. Multiple long-term conditions (previously known as multimorbidity), polypharmacy and ‘geriatric syndromes’, such as frailty, falls and delirium, all drive increased hospital admissions and uptake of healthcare services by this population [3].

Thus, as the population ages, doctors are having to manage patients who are more complex than ever before. Yet, with just one consultant geriatrician per 8000 of the UK population aged over 65, older patients are being cared for in nonspecialist areas, occupying approximately two-thirds of inpatient hospital beds [4]. This demographic emergency necessitates two urgent changes to our healthcare systems: firstly, we need to expand the provision of specialist geriatric care through investment and recruitment [5, 6] and, secondly, we need to upskill the entire workforce in the generalist principles required to care appropriately for older people with complex needs. Undergraduate medical education offers a singular opportunity to address this. By inspiring and training future clinicians, prior to their specialisation, we can create a medical workforce furnished with the knowledge, skills and attitudes required to care for our ageing population.

The WHO advocates for the education and training of doctors as a means to address the challenges posed to healthcare by the ageing population. UK medical schools have previously been surveyed on their undergraduate teaching of ageing and geriatric medicine (2008, 2013), which was found to be lacking [7, 8]. Despite improvements between 2008 and 2013, such as formalisation of assessment methods and an increase in the number of teaching hours, teaching on geriatrics still only accounted for, on average, 56 hours of teaching in an entire 4–6 -year medical degree [8].

In the intervening decade, much has progressed in geriatric medicine that has since been reflected in the updated British Geriatrics Society (BGS) recommended undergraduate curriculum (2023) [9]. There has also been transformation in UK medical education, with the expansion of medical school and medical student numbers and the introduction of the nationally mandated and standardised ‘Medical Licensing Assessment’ (MLA) [10, 11]. It was timely, therefore, that we resurveyed UK institutions on their delivery of teaching in ageing and geriatric medicine, to identify exemplar practices and areas for further improvement. The aim of this, in tandem with updating the BGS recommended curriculum, is to build a model blueprint for undergraduate geriatric medicine education, serving as a benchmark for change in our educational culture and practice that will ultimately improve the care of older people.

## Methods

Between November 2021 and November 2022, an online questionnaire was distributed to all 35 General Medical Council (GMC)-registered medical schools in the UK via the authors’ combined networks and the BGS Education and Training Committee (ETC). Invitation emails were initially sent to the known institutional leads for geriatric medicine. Where this was not known, emails were sent to the head of the medical school (different job titles in different centres), with a request to forward onto the appropriate lead for geriatric medicine. The survey was also publicised by the BGS in their newsletters and on X (formerly Twitter).

The survey questions were the same for all respondents; however, the survey could be exited after only providing initial information, which was the only required part of the questionnaire (see the Supplementary Information). This first mandatory section of the questionnaire collected baseline contextual information about the individual and their institution, and summary information about any unit/module in geriatric medicine (collected in mixed format of multiple-choice questions and free-text questions). The second, optional, part of the survey collected details of the content, methodology, timing and duration of teaching and assessment of topics in ageing and geriatric medicine. This section of the survey was structured around the 2013 iteration of the BGS-recommended undergraduate curriculum [12], so the data collection mirrored that of previous surveys and allowed results to be compared (2008 and 2013).

Data has been analysed and is presented using simple descriptive statistics. During data analysis, as with the interpretation of previous national survey results, a non-response for an individual domain within an otherwise completed questionnaire was taken to indicate that a domain was not taught or examined.

## Results

### Response rate

There were 35 GMC-recognised medical schools in the UK at the time of survey distribution. Of these, 26 schools provided a full survey response. One school provided an incomplete response to the survey whereby limited information regarding the overall teaching structure was provided, but in-depth breakdown of topics was left blank. A further four schools that did not complete the survey were followed up via email and provided a more informal response, giving overview of their geriatric teaching. Five schools did not participate. All the responding schools (*n* = 30) reported some teaching on ageing and/or geriatric medicine.

### Module overview

There was a spread of geriatric medicine teaching throughout undergraduate medical courses, as presented in Table 1. Multiple schools described a longitudinal weaving of topics related to ageing and geriatric medicine throughout various years. Of those schools that did have dedicated modules in geriatric medicine, there was variation in the length and timing of these (Table 2). Of those reporting a module length > 12 weeks, one school taught this as a dedicated geriatric medicine module, while the other two incorporated geriatric medicine into an integrated clerkship with other specialties.

**Table 1:** The number of medical schools teaching an aspect of geriatric medicine in each undergraduate year

Year taught	Number of schools
1	2
2	3
3	15
4	21
5	9
6	3

**Table 2:** The length of clinical geriatric medicine modules in weeks

Module length (weeks)	Number of schools (%)
<4	10 (33.3)
4–8	15 (50)
8–12	1 (3.3)
>12	3 (10)
Not reported	1 (3.3)

### Topic coverage

Twenty-six schools provided detailed information about coverage of topics drawn from the BGS recommended undergraduate curriculum (2013)—detailed in Table 3. Fourteen schools provided responses to all three iterations of the survey (2008, 2013 and 2021). Data on teaching and assessment changes across these topics between surveys is detailed in Table 4.

**Table 3:** Number (%) of medical schools teaching and examining in each topic in 2021–22

Topic	Taught, *n* (% of respondents)	Examined, *n* (% of respondents)
Anatomical and histological changes associated with ageing	17 (65)	14 (54)
Autonomy and mental capacity	24 (92)	21 (81)
Cerebrovascular disease and stroke	25 (29)	24 (92)
Comprehensive geriatric assessment	25 (96)	22 (85)
Continence	22 (85)	19 (73)
Contributions of community geriatrics	18 (69)	12 (46)
Delirium	25 (96)	26 (96)
Dementia	26 (100)	25 (96)
Demography and epidemiology of ageing	17 (65)	9 (35)
Depression	25 (96)	23 (88)
Effect of ageing on pharmacodynamics and pharmacokinetics	25 (96)	24 (92)
Elder abuse	16 (62)	13 (50)
Ethical and legal aspects of geriatric medicine	25 (96)	18 (69)
Falls	26 (100)	26 (100)
Heterogeneity	25 (96)	18 (69)
Interaction between health and social services	19 (73)	12 (46)
International classification of function	11 (42)	8 (22)
Osteoporosis	24 (92)	23 (88)
Parkinsonism	25 (96)	21 (81)
Pathology of ageing and age-associated disease	19 (73)	17 (65)
Physiology of ageing	20 (77)	15 (58)
Polypharmacy	25 (96)	23 (88)
Practise of safe prescribing	25 (96)	20 (77)
Pressure ulcers	20 (77)	15 (58)
Professional approach to the older person	23 (88)	20 (77)
Professions allied to medicine	25 (96)	19 (73)
Psychosocial theories of ageing	12 (46)	10 (38)
Rehabilitation	23 (88)	19 (73)
Research design	8 (31)	5 (19)
Stereotypes and ageism	22 (85)	17 (65)
Theories of ageing	19 (73)	14 (54)
Understanding associated services, e.g. palliative care	20 (77)	12 (46)

**Table 4:** The percentage change in topics taught and examined in 2013 versus 2021

Topic	Taught in 2013 (%)	Taught in 2021 (%)	Change (%)	Examined in 2013 (%)	Examined in 2021 (%)	Change (%)
Advance directives^a^	74	N/A	N/A	58	N/A	N/A
Ageing and pharmacology	95	96	+1	79	92	+13
Assessment scales in health^a^	84	N/A	N/A	68	N/A	N/A
Cellular ageing	68	65	−3	58	54	−4
Delirium	100	96	−4	84	96	+12
Dementia	100	96	−4	89	96	+7
Demographics	95	65	−30	79	35	−44
Elder abuse	68	62	−6	53	50	−3
Ethics	100	96	−4	79	88	+9
Falls	95	100	+5	85	100	+15
Incontinence	95	85	−10	74	73	−1
Mental capacity	100	92	−8	79	81	+2
Models of services	84	77	−7	68	46	−22
Osteoporosis	95	92	−3	79	88	+9
Parkinsonism	89	96	+7	79	81	+2
Physiology of ageing	84	77	−7	63	58	−5
Polypharmacy	100	96	−4	84	88	+4
Pressure ulcers	79	77	−2	58	58	+0
Social ageing	74	46	−28	63	38	−25
Stroke	100	96	−4	79	92	+13
Terminology and classification of health^a^	37	N/A	N/A	21	N/A	N/A

^a^Topics reframed or reworded in the 2021 survey and therefore not comparable to the 2013 survey. These changes are reflected in the updated BGS recommended undergraduate curriculum.

### Teaching and assessment methods

There was a spread of both formal and informal teaching methods across topics and a shift towards smaller group- or case-based teaching. For example, in 2013, the topic of dementia was taught in lecture format in 61% (11/18) of schools, with 28% combining this with a smaller-group teaching or seminar format. In contrast, in 2021, only 12% (3/26) taught this topic solely through lectures, with all others combining large-group teaching with small-group, ward-based or case-based learning.

We also observed a shift in assessment methods from summative examination with multiple-choice questions (MCQs) to a wide range of assessments for each topic. Schools often used several assessment methods for individual topics, such as MCQs, objective structured clinical examinations (OSCEs) and workplace-based assessments (WPBAs). For instance, in 2013, ‘Parkinsonism’ was assessed by 15 schools, 53% (8/15) solely through summative written or MCQ examination. In 2021, this had decreased to 10% (2/21), and 19 schools assessed it through a combination of summative examination (MCQs, written, OSCEs), WPBAs and clinical portfolio.

### Exemplar teaching practices

We noted several exemplar teaching practices detailed in the free-text section of the survey, where schools were asked to describe aspects of their curriculum that they deemed to be unique. Several examples of these are described below.

In both the 2013 and 2021 iterations of the surveys, schools were asked to describe any interdisciplinary teaching practices. In 2013, 11 schools reported an element of teaching combined with that of other healthcare students, such as student nurses, pharmacists or allied healthcare professionals. We found that this decreased to just 8 schools reporting interprofessional learning (IPL) in geriatric medicine in 2021-22. Examples of this include the University of Nottingham, which introduced a ‘simulated ward round’ with both medical and nursing students playing their respective roles in a geriatric medicine setting. The University of Oxford taught geriatric medicine within a longitudinal integrated clerkship, most notable for their interprofessional approach to teaching ‘challenging behaviour’ in dementia, involving a wide range of clinical educators from different healthcare professions, including general practitioners (GPs), geriatricians and mental health nurses. In addition to this, some teaching was provided in collaboration with nursing students from Oxford Brookes University.

King’s College London and the University of Bristol have both significantly increased the amount of teaching on geriatric medicine since previous surveys; King’s College London updated their curriculum in 2018, which now includes over 30 hours of new teaching in geriatric medicine, including a 4-week block in year 1 dedicated to ageing. Similarly, Bristol Medical School updated its curriculum in 2017 to incorporate an 18-week clerkship in geriatric medicine into the fourth year of study. Not only is Bristol unique for its extended duration dedicated to geriatric medicine, but also its approach to teaching includes both evidence-based and novel innovations [13].

Brighton and Sussex Medical School introduced a programme called ‘Time for Dementia’, where students meet a person living with dementia in their homes with their caregiver(s) several times over 2 years. This example demonstrates how medical education can expand into the community, still providing students with authentic learning opportunities in the care of older people [14, 15]. Similarly, Newcastle University reported innovative inclusion of older people’s voices in curriculum codesign and delivery, through its novel NUAGE programme [16].

## Discussion

Our survey has demonstrated progress in undergraduate geriatric medicine teaching in the UK compared to 2013. We found that many aspects of geriatric medicine remain widely taught in UK medical schools, and topics such as frailty syndromes are being examined more widely than they were previously. However, there has been a reduction in the teaching and examination of psychosocial, holistic and community-based topics compared to 2013, and the basic and translational science of ageing remains poorly covered. We have observed a shift towards small-group teaching methods and the usage of a broader range of assessment methods. These results are reassuring that medical schools are responding to the needs of the ageing population by improving teaching offerings in geriatric medicine to ensure that graduates are equipped to care for older people—yet, there remain key gaps and therefore room for further improvement, which we will now discuss.

There was a broad spread of responses from schools from all UK nations, with an 86% response rate for basic module information. The in-depth survey response rate was higher than those of previous iterations, at 74% compared to 64% and 63% in 2008 and 2013, respectively. This high response rate means that we can be confident that our results present a rounded snapshot of the teaching and assessment of geriatric medicine at UK medical schools. It must be noted that more GMC-registered medical schools have opened in the UK since the 2013 survey, which provides a larger sample size but does not account for an increase in the response rate. Survey distribution may have been more effective than previously, given that it was distributed through the BGS ETC directly to known connections in medical schools—while a similar approach was used for previous surveys, this was done through the committee for Training Programme Directors (postgraduate), as the ETC had not yet been formed. This may indicate that the BGS as a body is better connected than previously, or that engagement with the BGS has improved. It may also indicate that more geriatricians are involved in medical education—certainly, in the past the quantity and quality of undergraduate education in geriatric medicine have been linked to the presence of geriatricians in university faculties [17]. Additionally, given that we have observed an overall improvement in teaching offering, it was more likely that medical schools would respond.

Our survey demonstrated that several topics were widely taught and examined. Twenty-five schools that provided a full survey response (96%) taught the topics of ‘dementia’, ‘delirium’, ‘falls’ and ‘pharmacology in ageing’, while 24 (92%) taught ‘stroke’, ‘polypharmacy’, ‘ethics’ and ‘parkinsonism’. These percentages are comparable to the 2013 survey, and to other topic-specific national surveys of teaching on delirium and dementia [18, 19]. If we incorporate these topics under the umbrella of ‘frailty syndromes’, we are able to demonstrate an improvement in teaching compared to a 2021 survey of frailty education by Winter et al. [20]. A recent Europe-wide study demonstrated that 40% of older people presenting to emergency departments have at least moderate frailty, and therefore the increased teaching of frailty-related syndromes is encouraging and will aid to equip the future workforce with the knowledge and skills required to manage these presentations [21].

Topics with less coverage were similar to previous surveys and included ‘international classification of function’, ‘physiology of ageing’, ‘social theories of ageing’, ‘elder abuse’ and ‘demographics’. In fact, our survey demonstrated a reduction in the teaching and examination of these topics compared to 2013. The lack of coverage of elder abuse amongst UK medical schools is in keeping with the 2008 survey, and should continue to raise concern [7], as elder abuse continues to be a poorly recognised and monitored issue in UK healthcare [22, 23]. It is possible that the incorporation of geriatric medicine into an integrated clerkship by several medical schools has meant that these topics have been left out due to resource and time constraints. Moreover, it is possible that the true numbers may be higher if these topics were taught earlier in the programmes, unbeknownst to the clinical educators responding to the survey—this is certainly possible given that these topics feature in the MLA content map as ‘areas of professional knowledge’ and ‘clinical and professional capabilities’, rather than in ‘areas of clinical practice’. However, developing and evaluating strategies to teach about elder abuse in health profession education remains a priority area for future research.

There was a clear shift away from didactic lecture-based learning formats and a widespread increase in small-group, problem-based learning (PBL) and case-based learning (CBL). Both PBL and CBL have been demonstrated to provide an effective and engaging format for both students and teachers [24, 25]. This shift is in keeping with the overall change in undergraduate medical education in the UK, with nearly all schools now using a mixed-methods approach of integrated or small-group teaching, and may also explain why teaching in geriatric medicine is weighted towards the later years of medical degrees where these clinical frameworks for medical education are in more frequent employ (Table 1) [26]. Challenges associated with teaching frailty have been noted previously, with students often struggling to conceptualise the complexity of frailty; however, Nimmons *et al*. demonstrated that clinically relevant small-group teaching can improve students’ confidence, knowledge and attitudes towards frailty [27]. Additionally, we know that lecture-based, didactic teaching does not correlate with positive attitude formation in medical education [14]. Thus, the challenges of teaching complexity and frailty may be better addressed within clinical frameworks, such as in PBL and CBL. Therefore, this shift is a positive one for our specialty, as is the shift towards a broader range of assessment methods, including clinical assessments (workplace-based or portfolio-based), which encourage students to contextualise and apply their learning in authentic and relevant ways. We suggest that future research should evaluate the impact of technology on the authenticity of clinical learning and assessment, and explore the facilitators and barriers to more formally involving expert patients and caregivers as health profession educators to enrich educational experiences [28].

A limitation of our survey is that while we can accurately capture the taught elements of curricula in medicine of ageing, any informal teaching in the clinical environment can only be estimated. Despite most schools reporting a high number of topics covered in formal teaching, several reported integration of geriatric medicine teaching within a general internal medicine clerkship. In many of these cases, students are randomly allocated a selection of medical specialties and, therefore, may or may not gain experience in geriatric medicine. By not surveying medical students themselves, we are unable to accurately assess the exposure to geriatric medicine within an integrated clerkship. As mentioned above, learning within a clinical framework significantly improves students’ understanding, and therefore missed opportunities for ward placements in geriatric medicine (through random allocation) may be negatively affecting learning.

Disappointingly, exposure to IPL was low. IPL is used nationally and internationally as a method of improving healthcare service and delivery, stipulated by the WHO [29]. It has been demonstrated to be an effective method for healthcare education, by allowing learning in a context that reflects future clinical practice [30]. The reasons for the low use of IPL are several. The key in this context may be the effects of the COVID-19 pandemic, as several schools described a switch to online learning and reduced face-to-face teaching during this time—we would hope that this may improve with resurvey and/or with the introduction of novel educational technologies. Additionally, the traditional perceived barriers to implementation of IPL within healthcare are still present. A major logistical barrier is fragmentation of healthcare and health profession education, with lack of leadership and coordination between organisations and the need for formal agreements to support planning and delivery [31]. Resources are often an obstacle, given that significant funding is required to enable the curriculum development, faculty remuneration and location of these initiatives [31]. Finally, perceived hierarchies within healthcare have been demonstrated to hinder how IPL approaches are operationalised in practice [32].

We acknowledge the limitations of our study. Firstly, the survey is only able to capture data in a cross-section of time, and we will have missed any curricular innovation since survey close. Given the introduction of the MLA in the academic year 2024–25, it could be that universities have updated their curricula since surveying—however, this is unlikely, given the long lead time required for curriculum transformation. Moreover, in our survey analysis, a nonresponse was taken to mean that a topic was not taught or assessed, whereas this may not represent the truth if the questionnaire was incomplete for other reasons (e.g. respondents’ lack of knowledge, rather than a true lack of teaching). Additionally, there is response bias in that schools with more robust teaching in geriatric medicine are more likely to respond to a survey and describe their exemplar practices. We minimised this bias by achieving a good response rate. Finally, only GMC-registered medical schools were contacted to complete the survey, while five other medical schools were active in the UK at that time, awaiting their GMC approval. These schools often have innovative curricula, and we may be missing out on many exemplar practices by not surveying them. In future data collection, it would be useful to specifically target data collection from newer medical schools. We recommend, therefore, that this survey be repeated every 5 years to capture new schools and changes in practice, as per the timeline of previous national surveys in 2008 and 2013. We also suggest that the survey could be supplemented with a qualitative exploration of the facilitators and barriers to exemplar teaching methods, to maximise transferability.

## Conclusion

Undergraduate geriatric medical education in the UK continues to improve; however, there is still opportunity for further progress. Teaching on frailty and frailty syndromes is rightfully high; however, there is still significant scope to improve the teaching on the underlying pathophysiology of ageing and elder abuse remains undertaught. There has been a positive shift towards smaller-group teaching and teaching/assessment within clinical frameworks, which may improve students’ ability to understand and conceptualise complexity within geriatric medicine. There is significant scope to improve IPL within geriatric medicine in order to more accurately reflect the clinical environment. We hope that, alongside the BGS recommended undergraduate curriculum [9], this study offers insight into teaching and assessment practices in geriatric medicine, most importantly highlighting important gaps for targeted improvement.

## Supplementary Material

aa-25-0476-File002_afaf216

## References

[1] World Health Organisation . Ageing and Health 2022. https://www.who.int/news-room/fact-sheets/detail/ageing-and-health (5 August 2025, date last accessed).

[2] Public Health England . Ch. 3: Trends in morbidity and risk factors. In: Health Profile for England, 2018. 2018. Available from: https://www.gov.uk/government/publications/health-profile-for-england-2018/chapter-3-trends-in-morbidity-and-risk-factors.

[3] Inouye S, Studenski S, Tinetti M et al. Geriatric syndromes: clinical, research and policy implications of a core geriatric concept. J Am Geriatr Soc 2008;55:780–91. 10.1111/j.1532-5415.2007.01156.x. PMC2409147 17493201

[4] British Geriatrics Society . Protecting the Rights of Older People to Health and Social Care*.* https://www.bgs.org.uk/policy-and-media/protecting-the-rights-of-older-people-to-health-and-social-care (5 August 2025, date last accessed).

[5] British Geriatrics Society . The Case for More Geriatricians: Strengthening the Workforce to Care for an Ageing Population. https://www.bgs.org.uk/MoreGeriatricians (5 August 2025, date last accessed).

[6] British Geriatrics Society . #ChooseGeriatrics Campaign. https://www.bgs.org.uk/choose (5 August 2025, date last accessed).

[7] Gordon A, Blundell A, Gladman J et al. Are we teaching our students what they need to know about ageing? Results from the UK National Survey of Undergraduate Teaching in Ageing and Geriatric Medicine. Age Ageing 2010;39:385–8. 10.1093/ageing/afq011. 20176711

[8] Gordon A, Blundell A, Dhesi J et al. UK medical teaching about ageing is improving but there is still work to be done: the second National Survey of Undergraduate Teaching in Ageing and Geriatric Medicine. Age Ageing 2014;43:293–7. 10.1093/ageing/aft207. 24375323 PMC3927775

[9] Pearson GME, Winter R, Blundell A et al. Updating the British Geriatrics Society recommended undergraduate curriculum in geriatric medicine: a curriculum mapping and nominal group technique study. Age Ageing 2023;52. 10.1093/ageing/afac325. PMC9902152 36746388

[10] Lewis J . The Cap on Medical and Dental Student Numbers in the UK. https://commonslibrary.parliament.uk/research-briefings/cbp-9735/ (5 August 2025, date last accessed).

[11] General Medical Council . Medical Licensing Assessment. https://www.gmc-uk.org/education/medical-licensing-assessment (23 October 2023, date last accessed).

[12] Gordon A *.* The British Geriatrics Society Recommended Curriculum in Geriatric Medicine for Medical Undergraduates. https://www.bgs.org.uk/resources/the-bgs-recommended-curriculum-in-geriatric-medicine-for-medical-undergraduates (5 August 2025, date last accessed).

[13] Pearson GM, Welsh T, Pocock LV et al. Transforming undergraduate education in geriatric medicine: an innovative curriculum at Bristol Medical School. Eur Geriatr Med 2022;13:1487–91. 10.1007/s41999-022-00690-w. 36071347 PMC9451112

[14] Daley S, Hebditch M, Jones C et al. Time for dementia: quantitative evaluation of a dementia education programme for healthcare students. Int J Geriatr Psychiatry 2023;38:e5922. 10.1002/gps.5922. 37208949

[15] Banerjee S, Jones C, Wright J et al. A comparative study of the effect of the time for dementia programme on medical students. Int J Geriatr Psychiatry 2021;36:1011–9. 10.1002/gps.5532. 33686788 PMC9291285

[16] Forster J, Tullo E, Wakeling L et al. Involving older people in inclusive educational research. J Aging Stud 2021a;56:100906. 10.1016/j.jaging.2020.100906. 33712091

[17] Keller I, Makipaa A, Kalenscher T et al. *Global Survey on Geriatrics in the Medical Curriculum*. Geneva: World Health Organization, 2002.

[18] Fisher JM, Gordon AL, MacLullich AM et al. Towards an understanding of why undergraduate teaching about delirium does not guarantee gold-standard practice – results from a UK national survey. Age Ageing 2014;44:166–70. 10.1093/ageing/afu154. 25324329 PMC4601531

[19] Tullo E, Gordon AL. Teaching and learning about dementia in UK medical schools: a national survey. BMC Geriatr 2013;13:29. 10.1186/1471-2318-13-29. 23537386 PMC3614474

[20] Winter R, Al-Jawad M, Wright J et al. What is meant by “frailty” in undergraduate medical education? A national survey of UK medical schools. Eur Geriatr Med 2021;12:355–62. 10.1007/s41999-021-00465-9. 33651346 PMC7990827

[21] European Taskforce on Geriatric Emergency Medicine (ETGEM) collaborators . Prevalence of Frailty in European Emergency Departments (FEED): an international flash mob study. Eur Geriatr Med 2024;15:463–70. 10.1007/s41999-023-00926-3. 38340282 PMC10997678

[22] House of Lords . Domestic Abuse of Older People. 2021. https://lordslibrary.parliament.uk/domestic-abuse-of-older-people/

[23] Stephens C, Mays N, Issa R et al. Elder abuse in the UK: out of the shadows and on to the agenda. BMJ 2021;375:n2828. 10.1136/bmj.n2828. 34819276

[24] Thistlethwaite JE, Davies D, Ekeocha S et al. The effectiveness of case-based learning in health professional education. A BEME systematic review: BEME Guide No. 23. Med Teach 2012;34:e421–44. 10.3109/0142159X.2012.680939. 22578051

[25] Trullàs JC, Blay C, Sarri E et al. Effectiveness of problem-based learning methodology in undergraduate medical education: a scoping review. BMC Med Educ 2022;22:104. 10.1186/s12909-022-03154-8. 35177063 PMC8851721

[26] British Medical Association . Courses at Medical School. 2024. https://www.bma.org.uk/advice-and-support/studying-medicine/becoming-a-doctor/courses-at-medical-school (5 August 2025, date last accessed).

[27] Nimmons D, Pattison T, O’Neill P. Medical student attitudes and concepts of frailty and delirium. Eur Geriatr Med 2018;9:45–50. 10.1007/s41999-017-0018-y. 34654279

[28] Teodorczuk A, Abdool PS, Yap CX et al. New horizons in undergraduate geriatric medicine education. Age Ageing 2024;53:afae050. 10.1093/ageing/afae050. 38688484

[29] World Health Organization . *Framework for Action on Interprofessional Education and Collaborative Practice*. Geneva: WHO, 2010. 21174039

[30] Reeves S, Fletcher S, Barr H et al. A BEME systematic review of the effects of interprofessional education: BEME Guide No. 39. Med Teach 2016;38:656–68. 10.3109/0142159X.2016.1173663. 27146438

[31] Sunguya BF, Hinthong W, Jimba M et al. Interprofessional education for whom? Challenges and lessons learned from its implementation in developed countries and their application to developing countries: a systematic review. PloS One 2014;9:e96724. 10.1371/journal.pone.0096724. 24809509 PMC4014542

[32] Steinert Y . Learning together to teach together: interprofessional education and faculty development. J Interprof Care 2005;19:60–75. 10.1080/13561820500081778. 16096146

