# Supplementary material for: What are we teaching UK medical students about ageing and geriatric medicine? Results of the third British Geriatrics Society national curriculum and teaching survey
Source: Age Ageing. 2025 Aug 11;54(8):afaf216. doi: 10.1093/ageing/afaf216 (PMC12342901; doi:10.1093/ageing/afaf216)
Supplement: aa-25-0476-File002_afaf216 [file aa-25-0476-file002_afaf216.docx]

**Welcome**

Welcome to the Third National Survey of Undergraduate Teaching in Ageing and Geriatric Medicine.

Taking part in our study will involve completing a **one-off online questionnaire**, which will collect **details of the content, methodology, timing and duration of teaching in geriatric medicine at your institution**. We estimate that this will take no more than 30 minutes to complete.

**This survey is based around the British Geriatrics Society's recommended curriculum for medical undergraduates**. This was derived through a series of consensus meetings amongst geriatricians, sociologists, biogerontologists and gerontechnologists regarding what should be taught to medical undergraduates about ageing. **We have mapped the agreed learning outcomes to the GMC Outcomes for Graduates document and a curriculum map can be provided on request**.

[Insert details here about funding and endorsements]

**If you have any questions, please contact [project lead name] via email:** [project lead email]

Thank you in anticipation for your assistance.

**Your details & Institutional details**

#### Please provide us with some basic information regarding you. This will allow us to contact you to clarify any areas of uncertainty surrounding your responses, and to provide you with details of the medical student survey. We also aim to provide individualised, anonymised data comparing your school's performance with that of others. All data will be handled confidentially, and your name and institution will not be identified in any publications.

- Name
- Job title
- Email address
- Medical School (select from drop down list)
- Type of course(s) offered at your institution (select all that apply)
  - Undergraduate with foundation year (6 years)
  - Undergraduate (5 years)
  - Graduate-entry (4 years)
  - Other (please specify)
- Type of curriculum delivered at your institution (as defined [here](https://www.bma.org.uk/advice-and-support/studying-medicine/becoming-a-doctor/courses-at-medical-school) by the British Medical Association)
  - Traditional (pre-clinical/clinical)
  - Integrated/systems-based
  - Case-based learning
  - Problem-based learning
  - Enquiry-based learning
  - Other (please specify)
- Does your medical school have a unit/module/clerkship on geriatric medicine? By this we mean a unit of teaching dedicated, or partly dedicated, to clinical attachments and teaching in geriatric medicine. Please answer ‘yes’ if there is a unit of teaching covering multiple specialties, of which geriatric medicine is one. This question does not refer to teaching themes that include aspects of ageing, spanning multiple years of the curriculum.
  - If yes: In which year(s) does this unit of teaching take place? (select all that apply)
    - Year 1
    - Year 2
    - Year 3
    - Year 4
    - Year 5
    - Year 6
  - If yes: How long is this unit of teaching in geriatric medicine? (If the unit of teaching covers multiple specialties, please estimate how much time is dedicated to geriatric medicine).
    - 1-4 weeks
    - 4-8 weeks
    - 8-12 weeks
    - 12-18 weeks
    - >18 weeks
  - If yes: Optional freetext box to provide more detail
  - If no: Are there any units of teaching or teaching themes that incorporate ageing or geriatric medicine? Please explain in as much detail as possible, including timing (year group) and duration of any teaching.
    - Free-text

**Curriculum/Teaching questionnaire**

The learning outcomes from the [BGS recommended undergraduate curriculum](https://www.bgs.org.uk/resources/the-bgs-recommended-curriculum-in-geriatric-medicine-for-medical-undergraduates) have been divided into 5 sections for the purposes of this survey.

1. Foundations of Ageing and Geriatric Medicine
2. Specific age-related conditions
3. Multidisciplinary team working
4. Prescribing in Geriatric Medicine
5. Ethicolegal Aspects of Geriatric Medicine
6. Research in Ageing and Geriatric Medicine

We have worked hard to make the questionnaire easy to use. We understand, however, that curricula vary from school to school. To allow for this variability, we have provided a large "free text" box at the bottom of each section. Some suggestions as to how you might use this box include:

- Where teaching is delivered by more than one professional group on more than one occasion or is assessed in various ways you may wish to list specific details.
- Where you teach some but not all of a subject you may wish to provide clarification regarding this.
- Where peculiarities of your local curriculum make it difficult to answer concisely you may wish to provide some supporting text.

The survey aims to concentrate on formal, timetabled teaching which is delivered to all undergraduates at your school. We are not particularly concerned, in this instance, with teaching which is delivered at some centres by some teachers but is not replicated across your course.

The following sections of the questionnaire will follow the same structure/content.

For each learning outcome (or groups of learning outcomes) in turn, participants will be asked:

- How is it taught? (select all that apply)
  - Not taught
  - Large-group teaching (lecture or similar)
  - Small-group teaching (tutorial or similar)
  - Computer-aided learning
  - Library/Book-based learning
  - Case/Problem/Enquiry-based learning
  - Simulation
  - Ward-based teaching (formal)
  - Ward-based teaching (informal)
  - Other (please specify)
  - I’m unsure/I don’t know
- In which year is it predominantly taught? (select all that apply)
  - Year 1
  - Year 2
  - Year 3
  - Year 4
  - Year 5
  - Year 6
  - I’m unsure/I don’t know
- Who teaches it? (select all that apply)
  - Consultant Geriatricians
  - Specialist Trainees in Geriatric Medicine (SpRs/Clinical Fellows)
  - Old-age Psychiatrists
  - General Physicians
  - General Practitioners
  - Nurses
  - Allied-healthcare Professionals
  - Non-clinical teachers
  - Others (please specify)
  - I’m unsure/I don’t know
- How much time is devoted to it?
  - <1 hour
  - 1-4 hours
  - 4-10 hours
  - >10 hours
  - I’m unsure/I don’t know
- What is the predominant mode of assessment? (select all that apply)
  - Not assessed
  - Summative assessment – MCQ
  - Summative assessment – written
  - Summative assessment – OSCE
  - Formative assessment – written
  - Formative assessment – presentation
  - Formative assessment – clinical portfolio
  - Objective long case
  - Workplace-based assessment (CBD, mini-CeX)
  - Other (please specify)
  - I’m unsure/I don’t know
- Optional freetext answer box for any further information

**Learning Outcomes by Section:**

**Foundations of Ageing and Geriatric Medicine**

- **1a. Students should be able to** maintain a professional approach to the older person
- **1**b. **Students should be able to** give consideration to various myths and stereotypes related to older people and c. advocate against ageism and recognise that it can affect the optimal care of older patients
- **1**d. **Students should be able to** recognise the heterogeneity of older persons and that each person needs to be viewed as an individual
- **3a. Students should be able to describe / define** biochemical, molecular, cellular, genetic theories of ageing
- **3**b. **Students should be able to describe / define** the anatomical and histological changes associated with ageing
- **3**c. **Students should be able to describe / define** the pathology associated with normal ageing and age associated disease processes
- **3**d. **Students should be able to describe / define** the physiology of ageing
- **9**c. **Students should be able to describe** psychosocial theories of ageing
- **8a. Students should be able to** define the components of the International Classification of Function (ICF) and discuss its advantages and disadvantages in comparison to previously used classifications
- **8**b. **Students should be able to** define Comprehensive Geriatric Assessment (CGA) and list its main domains to incorporate medical, psychological, social, functional and environmental, and c. describe the process of Comprehensive Geriatric Assessment, including initial assessment, establishment of a problem list, management plan, goals and iteration. Describe the role of the multidisciplinary team in this process.

**Specific age-related conditions**

3f. the diagnosis, pathophysiology, management and preventative strategies for specific disease processes:

- Dementia
- Delirium
- Depression
- Continence
- Osteoporosis
- Falls
- Parkinsonism & movement disorders
- Pressure ulcers
- Cerebrovascular disease and stroke

**Multidisciplinary team working**

- **2a. Students should be able to** describe the contributions of the following professions allied to medicine: Occupational therapy, Physiotherapy, Nurses, Dieticians, Speech & language therapists, Social workers, and b. explain how these professions can work together effectively as part of a multidisciplinary team.
- **5a. Students should be able to** recognise the limitations of hospital-based care and value the contribution of Community Geriatrics in adequate assessment and management of patients
- **5**b. **Students should be able to** describe the concept of Rehabilitation
- **5**c & 9b. **Students should be able to** define the interaction between health and social services in the provision of long-term care for older adults and describe the following services; NHS continuing care, residential home care, nursing home care, community care at home, community nursing care, community matron service, intermediate care at home, residential intermediate care, interim care
- 8d. **Students should be able to** define the following specialties and relate their contribution to the care of older adults; continence services, falls services, intermediate care, old age psychiatry, orthogeriatrics, palliative care, stroke medicine.

**Prescribing in Geriatric Medicine**

- 3e. **Students should be able to describe / define** the effect of ageing upon pharmacodynamics and pharmacokinetics
- **4a. Students should be able to describe the concept of** polypharmacy
- **4**b. **Students should be able to describe the concept of** the practice of safe prescribing in older adults, taking account of differing physiology, drug interactions and multiple pathologies

**Ethicolegal Aspects of Geriatric Medicine**

- **6a-e. Students should be able to describe ethical and legal issues including:** advance directives, euthanasia and assisted suicide, safeguarding, withdrawal and withholding of medical treatment, cardiopulmonary resuscitation decisions.
- **7a. Students should be able to describe** the principles of autonomy, mental capacity to make decisions and the concept of “best interests”, and b. the legislation in each jurisdiction which outlines and protects these principles.
- 10. Students should be aware of the issue of elder abuse (physical, psychological and financial)

**Research in Ageing and Geriatric Medicine**

- **9a. Students should be able to describe** recent and predicted trends in demography and epidemiology of ageing
- 11a. Students should be able to describe how to design research where the findings are applicable to older people including:
- issues of sampling and generalisability
- inclusion of participants with functional and cognitive impairment and the associated ethical issues
- issues of measurement and measurement bias due to ceiling and floor effects in the older population
- And 11b. discuss the generalisability of existing research studies to frail older people through consideration of these issues.

**Section 7: Innovations and Changes in Teaching**

- Does your institution use interdisciplinary teaching as part of their teaching on ageing and geriatric medicine? By interdisciplinary teaching we mean medical student and other health sciences students (e.g. dentistry, nursing, physiotherapy, pharmacy) being taught side-by-side in the same session.
  - Yes
  - No
  - Unsure
  - If yes: Optional freetext answer box to provide details)
- We would like to take this opportunity to gather data on exemplary teaching practices related to ageing and geriatric medicine. If there is any programme within your school of which you are particularly proud and which you would like to share as a model of innovation, please tell us about it below.
  - Optional freetext answer box
- Are there any planned changes to how ageing and geriatric medicine are taught in your medical school? If so, please outline the planned changes and the rationale for these.
  - Optional freetext answer box

**Closing page**

Thank you for your help in completing this survey.

If you have any questions, please contact [project lead name] via email: [project lead email]. Should you have any concerns about this research please contact [research governance email].
